# Implementation of quality management systems and progress towards accreditation of National Tuberculosis Reference Laboratories in Africa

**DOI:** 10.4102/ajlm.v6i2.490

**Published:** 2017-03-31

**Authors:** Heidi Albert, Jean de Dieu Iragena, Kekeletso Kao, Donatelle Erni, Teferi Mekonen, Philip C. Onyebujoh

**Affiliations:** 1 FIND, Cape Town, Western Cape, South Africa; 2 World Health Organisation, African Region Country Office, Brazzaville, Congo; 3 FIND, Campus Biotech, Geneva, Switzerland; 4 African Society for Laboratory Medicine, Addis Ababa, Ethiopia; 5 World Health Organisation, African Region Country Office, Harare, Zimbabwe

## Abstract

**Background:**

Laboratory services are essential at all stages of the tuberculosis care cascade, from diagnosis and drug resistance testing to monitoring response to treatment. Enabling access to quality services is a challenge in low-resource settings. Implementation of a strong quality management system (QMS) and laboratory accreditation are key to improving patient care.

**Objectives:**

The study objective was to determine the status of QMS implementation and progress towards accreditation of National Tuberculosis Reference Laboratories (NTRLs) in the African Region.

**Method:**

An online questionnaire was administered to NTRL managers in 47 World Health Organization Regional Office for Africa member states in the region, between February and April 2015, regarding the knowledge of QMS tools and progress toward implementation to inform strategies for tuberculosis diagnostic services strengthening in the region.

**Results:**

A total of 21 laboratories (43.0%) had received SLMTA/TB-SLMTA training, of which 10 had also used the Global Laboratory Initiative accreditation tool. However, only 36.7% of NTRLs had received a laboratory audit, a first step in quality improvement. Most NTRLs participated in acid-fast bacilli microscopy external quality assurance (95.8%), although external quality assurance for other techniques was lower (60.4% for first-line drug susceptibility testing, 25.0% for second-line drug susceptibility testing, and 22.9% for molecular testing). Barriers to accreditation included lack of training and accreditation programmes. Only 28.6% of NTRLs had developed strategic plans and budgets which included accreditation.

**Conclusion:**

Good foundations are in place on the continent from which to scale up accreditation efforts. Laboratory audits should be conducted as a first step in developing quality improvement action plans. Political commitment and strong leadership are needed to drive accreditation efforts; advocacy will require clear evidence of patient impact and cost-benefit.

## Introduction

The burden of tuberculosis in Africa remains high. The World Health Organization (WHO) reported 1 342 000 tuberculosis cases in the region in 2014; 28% of the global caseload. The region suffers from the highest per-capita burden; 281 cases per 100 000 population, more than double the global average. Despite meeting the Millennium Development Goal target of a falling tuberculosis incidence rate, the African region failed to meet the targets for 50% reduction in tuberculosis prevalence and mortality. Furthermore, the attainment of these targets varied among countries, with 40 countries achieving the Millennium Development Goal tuberculosis incidence target and only 18 countries achieving the 50% reduction in tuberculosis mortality target. Rates of multi-drug resistant tuberculosis vary across the continent, with average rates of 2.1% (0.5–3.7) among new cases and 11% (6.7–16) among previously-treated cases.^1^

High-quality laboratory services are an essential component of all stages of the tuberculosis care cascade, from diagnosis and drug resistance testing to monitoring response to treatment.^2,3^ However enabling access to quality tuberculosis diagnostic services for populations in need is a major challenge in low-resource settings. The End TB Strategy calls for universal access to drug susceptibility testing (DST).^4^ However, the WHO reported that only 6.4% of new bacteriologically-confirmed tuberculosis cases and 33% of previously-treated cases received DST in 2014.^1^

Laboratory services on the continent are known to suffer many challenges, including poor infrastructure, inadequate human resource capacity, and weak underlying health systems.^3^ A number of recent regional initiatives have emphasised the need for strengthening quality systems of laboratories in the region, including the World Health Organization (WHO) Regional Office for Africa 58th session (AFR/RC58/R6, Yaoundé, September 2008),^5^ the Maputo Declaration (January 2008),^6^ the Kigali Declaration on Strengthening Laboratory Management Towards Accreditation (July 2009),^2^ and the African Society for Laboratory Medicine’s Ministerial Call for Action (December 2012).^7^

A strong laboratory quality management system (QMS) is critical to ensuring the quality of testing. A weak QMS may result in laboratory errors that can lead to both over- and under-diagnosis of tuberculosis, interruptions in service, and delayed reporting of results, leading to a negative impact on patient care. Failure to meet minimum safety standards may put laboratory workers, patients and the community at risk.^8^ Such requirements can only be consistently met by concerted efforts to develop and maintain quality management systems within the tuberculosis laboratories. Accreditation is a formal process of recognition that appropriate and sufficient quality systems have been implemented. Accredited laboratories are recognised as meeting certain quality standards and having the necessary technical processes in place, as well as administrative systems needed to ensure quality results. Evidence from various high- and low-income settings^8,9,10,11^ demonstrates that the implementation of QMS and accreditation leads to a measurable improvement in the quality of services and increased patient safety due to a reduction in laboratory errors.

National Tuberculosis Reference Laboratories (NTRLs) play a critical role in ensuring quality tuberculosis diagnostic services throughout the whole diagnostic testing network, conducting advanced testing (such as liquid culture, first- and second-line DST), supervision of peripheral laboratories, external quality assurance, and conducting drug surveillance surveys and operational research. Quality improvement of NTRLs is a critical first step in building strong diagnostic networks to deliver quality laboratory services for patient care.

Accreditation of NTRLs was a specific goal in the Global Plan to Stop TB (2006–2015).^12^ The plan estimated that less than 5% of NTRLs globally were accredited by 2006, and a target of more than 50% NTRLs being accredited by 2015 was established. Despite some progress, the goal of 50% NTRLs being accredited remains unmet and, especially in the African Region, only a minority of NTRLs, or any other tuberculosis laboratories, have reached this standard. At the time of writing, NTRLs in South Africa, Botswana, Mozambique and Uganda (8% of NTRLs in the African Region) had achieved accreditation according to the ISO 15189 standard (South Africa National Accreditation Service, www.sanas.co.za and Instituto Portugues de Acreditacao, www.ipac.pt). Fifty-four per cent of countries in the African Region reported having a formal QMS toward achieving laboratory accreditation implemented at the NTRL in 2014.^1^ However, the extent of progress toward accreditation of individual laboratories remains unclear. Furthermore, only six NTRLs have received a formal external audit conducted by the African Society for Laboratory Medicine using the WHO’s Stepwise Laboratory Quality Improvement Process Towards Accreditation (SLIPTA) programme (Mekonen T, personal communication).

There are a number of key resources available to assist tuberculosis laboratories in developing and maintaining a QMS, which are reviewed in the Global Laboratory Initiative’s (GLI) recently-published *Guide for Providing Technical Support to TB Laboratories in Low- and Middle-income Countries*.^13^ Adherence to the ISO 15189:2012 international standard (www.iso.org) is used by regulating authorities and accreditation bodies for recognising the competence of medical laboratories. The WHO’s Laboratory Quality Management System provides a comprehensive overview of laboratory QMS and comes in the form of a modular training package and manual.^14^ GLI developed an online tuberculosis-specific tool to assist laboratories with accreditation preparedness (www.gliquality.org). SLIPTA is a stepwise monitoring and auditing framework developed by the WHO Regional Office for Africa, based on ISO 15189. Assessment using the SLIPTA checklist is scored and rated on a scale of one to five stars, with five stars being considered an indicator of readiness for international accreditation. Strengthening Laboratory Management Toward Accreditation (SLMTA) is a structured quality improvement programme, which teaches laboratory managers how to implement practical QMS in resource-limited settings (www.slmta.org). The programme includes a series of workshops and work-based improvement projects supported by site visits and mentoring, and impact of the programme is measured using the SLIPTA checklist.^15^ The Foundation for Innovative New Diagnostics found has developed a tuberculosis-specific version of SLMTA (TB-SLMTA), including a harmonised checklist based on SLIPTA and the GLI tool.^16^

GLI Africa, a partnership focused on strengthening tuberculosis diagnostic networks in the African Region,^17^ has established a programme aimed at supporting quality improvement and accreditation of NTRLs on the continent. As a first step in this initiative, a survey was developed to determine the current status of implementation of QMS at NTRLs, and the knowledge and use of various tools to assist in accreditation preparedness.

## Methods

### Survey development and dissemination

The aim of the survey was to gather baseline data on NTRL status regarding QMS and accreditation, to inform planning for support to countries working towards accreditation. The questionnaire was developed between December 2014 and February 2015 by a GLI Africa taskforce, which comprised key stakeholders, including the African Society for Laboratory Medicine, WHO Regional Office for Africa, GLI, the WHO Supranational Reference Laboratory (SRL) Uganda, the US Centers for Disease Control and Prevention, and FIND. Following finalisation of the questionnaire content in English, translations were made into French and Portuguese. The survey was disseminated via email from the WHO Regional Office for Africa to the WHO representatives/WHO liaison officers in 47 WHO African Region member states, containing links to the survey in the three languages. The survey was disseminated on 4 February 2015, along with an explanation of the purpose of the survey, and the closing date for responses was 17 February 2015. WHO country representatives were responsible for dissemination of the survey to the National Tuberculosis Control Programme (NTP) and NTRL managers. Organisations represented on the GLI Africa task force also followed up with NTP/NTRL managers in countries to encourage survey participation. Countries that did not respond by the deadline were contacted, either by one of the authors (J.I.) or by in-country consultants, to request a response. Respondees were given the option to respond via the online survey tool, or via email or fax. All responses were received by 25 April 2015.

### Survey questions

The survey consisted of questions in several key areas: (a) familiarity with various tools and approaches that can be used when implementing a QMS; (b) progress with implementing QMS in their laboratory; (c) data from internal or external QMS assessments; (d) programmes and partners supporting strengthening of QMS in their laboratory; and (e) challenges faced in strengthening QMS. Survey questions and possible responses are provided in full in Table 1.

**TABLE 1:** National Tuberculosis Reference Laboratory Accreditation Preparedness Survey questions

Questions		Possible responses
1	National Tuberculosis Reference Laboratory contact information	Name, institution, address, city, postal code and country
2	Head of National Tuberculosis Reference Laboratory contact information	Name, job title, email and phone number
3	Which of the tools for implementing QMS are you aware of? *Check any that apply*	Global Laboratory Initiative (GLI) Stepwise Process Towards TB Laboratory Accreditation Stepwise Laboratory Management Toward Accreditation (SLMTA) SLIPTA TB-SLIPTA harmonised checklist LQMS
4	Is your laboratory actively implementing a QMS towards accreditation using the GLI tool (http://www.gliquality.org/)?	Yes / No / Partially
5	If the answer to Q4 is yes, please indicate the phase of implementation (i.e. Phase 1, Phase 2, Phase 3 or Phase 4)	Phase 1 Phase 2 Phase 3 Phase 4
6	Has the National TB Reference Laboratory received an audit using the SLIPTA checklist?	Yes / No / Audit planned but not yet conducted
7	If the answer to Q6 is yes, what was the date of last audit?	
8	If the answer to Q6 is yes, what score and star grading did your laboratory achieve?	0 stars (0–141 points) 1 star (142–166 points) 2 stars (167–192 points) 3 stars (193–218 points) 4 stars (219–243 points) 5 stars (244–258 points)
9	Has your country appointed a SLIPTA focal person?	Yes / No
10	Have you included a budget and workplan for accreditation in your National Strategic Plan or Global Fund concept note?	Yes / No
11	If the answer to Q10 is yes, do you receive technical or financial support for accreditation?	Financial Technical Financial and technical None
12	If the answer to Q10 is affirmative, please name organisation providing support	Name Email Phone
13	If not working towards accreditation currently, what are the main reasons?	Lack of knowledge of staff Lack of accreditation programme Lack of advocacy Lack of training Lack of regular supervision Lack of staff motivation Lack of funds Other
14	Are staff from your TB laboratory participating in SLMTA or TB-SLMTA programme?	SLMTA TB-SLMTA SLMTA/TB-SLMTA None
15	Do you have a Formal Collaboration Agreement with a WHO Tuberculosis Supranational Reference Laboratory (SRL)?	Yes / No
16	If the answer to Q15 is yes, what is the name of the SRL	SRL Name
17	If the answer to Q15 is no, which SRL would you like to be linked to and have a formal agreement signed?	SRL Name
18	If the answer to Q15 is yes, what is the nature of support you receive?	Financial Technical Both None
19	Please list the type of support related to quality assurance or quality management system you received from each WHO SRL	
20	Does your laboratory participate in the following External Quality Assurance (EQA) programmes coordinated by WHO SRL(s)?	AFB smear microscopy First-line drug susceptibility testing Second-line drug susceptibility testing Other
21	Does your laboratory participate in the following External Quality Assurance (EQA) programmes coordinated by other providers?	AFB smear microscopy First-line drug susceptibility testing Second-line drug susceptibility testing Molecular TB and drug resistance testing Other
22	If your laboratory participates in EQA programme, please provide name (s) of EQA provider	EQA Provider name
23	Please provide any additional information related to your laboratory’s progress towards accreditation	
24	What is, in your view, the major barrier for implementing the Quality Management System in your laboratories using the available tools?	
25	Please provide SLIPTA focal person contact details in case your country has appointed that person	Full name Job title Email Phone

AFB, acid-fast bacilli; GLI, Global Laboratory Initiative; EQA, external quality assurance; LQMS, Laboratory Quality Management System; QMS, Quality Management System; SLIPTA, Stepwise Laboratory Quality Improvement Process Towards Accreditation; SLMTA, Strengthening Laboratory Management Toward Accreditation; SRL, Supranational Reference Laboratory TB, tuberculosis; WHO, World Health Organization.

### Data management

Data from the online survey were managed via the WHO extranet data system. Survey responses were exported from the WHO system in .csv format, and data analysis was conducted in Microsoft Excel (2013 version; Microsoft, Redmond, Washington, United States).

## Results

Responses were received from all 47 WHO African Region member states. A total of 49 responses from NTRLs were received; one per country except for Zimbabwe and Nigeria where each has two NTRLs which responded individually. All responses were submitted via the online survey tool.

### Knowledge of quality management system tools

A total of 93.9% (46/49) of NTRLs reported awareness of at least one of the QMS tools listed (GLI Stepwise Process Towards TB Laboratory Accreditation, WHO Laboratory Quality Management System Handbook, SLIPTA, SLMTA, and/or TB-SLMTA. Five NTRLs reported knowing all of the QMS tools mentioned, all of which had either achieved accreditation or were known to be actively working toward accreditation. Three laboratories reported having no knowledge of any of the tools; all three laboratories were from non-Anglophone countries. Overall, 69.2% of English-speaking NTRLs had knowledge of the GLI tool, compared with 60.0% for Portuguese-speaking NTRLs and 55.6% for French-speaking NTRLs.

### Progress with implementation of quality management system

**FIGURE 1:**
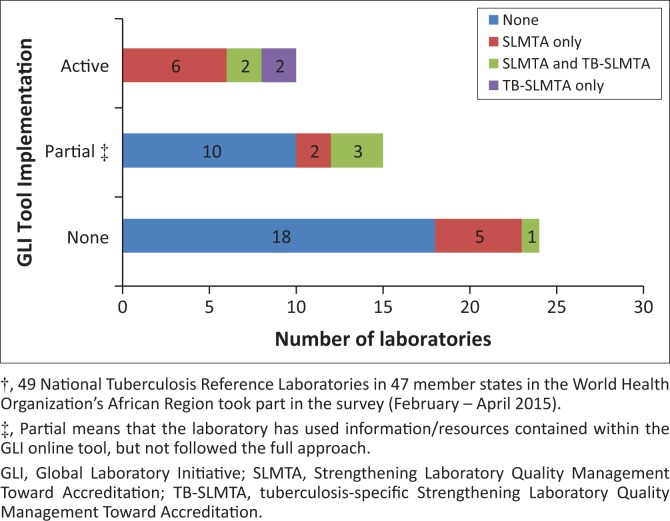
Implementation of GLI and SLMTA/TB-SLMTA approaches by National Tuberculosis Reference Laboratories in the World Health Organization’s African Region†

Twenty-one laboratories (42.9%) reported having staff trained by either SLMTA or TB-SLMTA; 13 (26.5%) by SLMTA, 6 (12.2%) had staff trained by both SLMTA and TB-SLMTA, while two (4.1%) reported staff receiving only TB-SLMTA training (Figure 1).

Ten laboratories reported actively implementing the GLI tool; two of which had already achieved accreditation and one laboratory was close to achieving accreditation at the time of the survey. Four additional laboratories reported partially implementing the GLI tool, all of which had been audited in 2013 or 2014 and received two, three, three and four SLIPTA stars levels. Twenty-one countries reported having a SLIPTA focal point appointed within the Ministry of Health.

All laboratories that reported using the GLI tool also reported having staff trained on SLMTA or TB-SLMTA. Two laboratories who reported actively implementing GLI and TB-SLMTA were from WHO SRLs that were already accredited but whose staff received TB-SLMTA training to build capacity for providing QMS support to other countries in the region.

**FIGURE 2:**
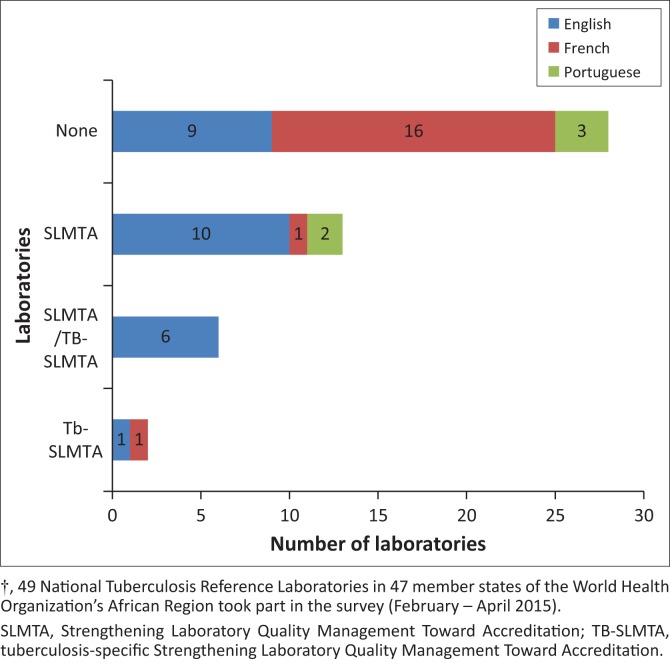
National Tuberculosis Reference Laboratories in the World Health Organization’s Africa Region† whose staff participated in SLMTA/TB-SLMTA training, according to country survey response language.

A higher proportion of Anglophone countries reported implementing QMS using the GLI tool and had received training by SLMTA or TB-SLMTA. 65.4% of countries that responded to the survey in English had staff who had participated in SLMTA and/or TB-SLMTA training, as opposed to 40.0% of Portuguese-speaking NTRLs and only 11.1% of French-speaking NTRLs. Implementation of a QMS using the GLI tool varied according to the language of the NTRLs, with 23.1% of English-speaking NTRLs, 20.0% of Portuguese-speaking NTRLs and 5.6% of French-speaking NTRLs reporting full use, and 30.8% of English-speaking NTRLs, 20.0% of Portuguese-speaking NTRLs and 22.2% of French-speaking NTRLs reporting partial use of the GLI tool (Figure 2).

### Audits

**FIGURE 3:**
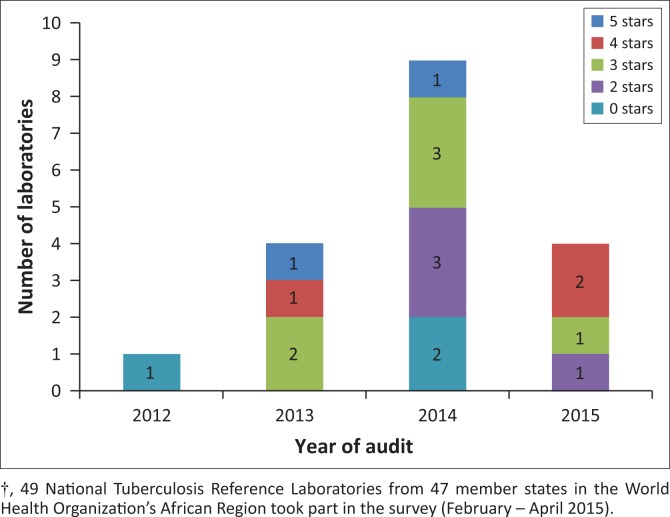
Auditing of National Tuberculosis Reference Laboratories in the World Health Organization’s African Region† using the Stepwise Laboratory Quality Improvement Process Towards Accreditation (SLIPTA) checklist, date of last audit and SLIPTA star grading achieved at last audit.

Eighteen NTRLs (36.7%) reported having had an audit conducted using the SLIPTA checklist. The date of the most recent audit and the audit score are shown in Figure 3. It was not documented whether these audits were internal or external and, if external, the identity of the auditing body. An additional four laboratories reported having planned audits that had not yet been conducted.

### External quality assurance

A total of 98.0% (48/49) of the NTRLs reported participation in at least one EQA programme, with 46 of these 48 sites (95.8%) being enrolled in acid-fast bacilli smear microscopy EQA. First-line DST was the next most common EQA in which NTRLs participated (60.4%), followed by second-line DST (25.0%) and molecular tuberculosis testing (22.9%). The most commonly-reported EQA providers were the WHO SRL network and the National Institute for Communicable Diseases (South Africa). Of the 20 NTRLs not enrolled in first-line DST EQA, 13 of these laboratories had links with an SRL.

### National Tuberculosis Reference Laboratories’ formal collaboration agreements with supranational reference laboratories

**FIGURE 4:**
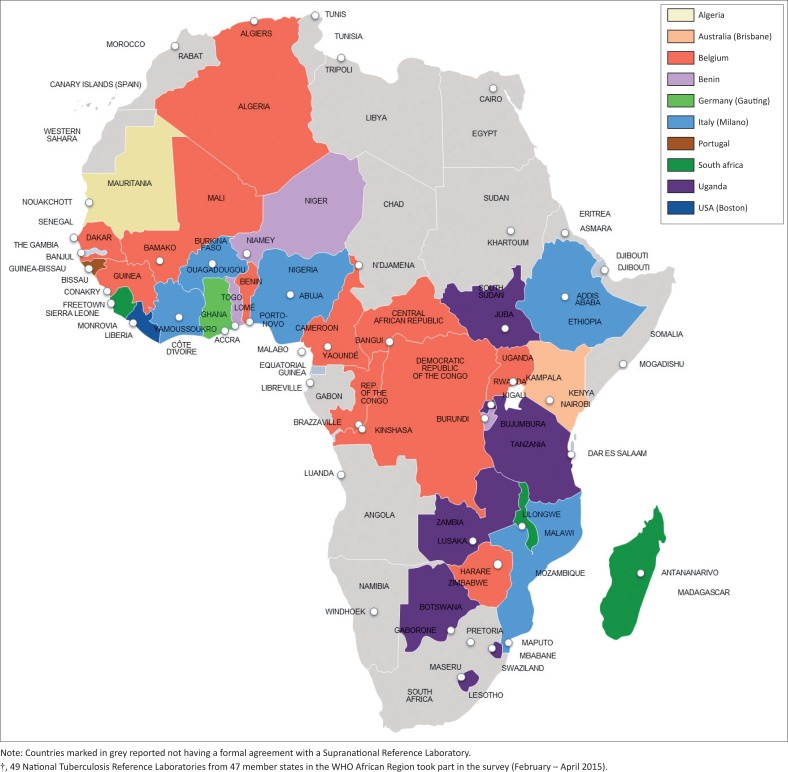
Collaboration agreements established between World Health Organization Supranational Reference Laboratories and National Tuberculosis Reference Laboratories (in the World Health Organization African Region†)

Forty (40/49; 81.8%) NTRLs reported having a formal agreement with an SRL. The majority of SRL support came from Belgium (30%), South Africa (18%), Uganda (18%) and Italy (12%) (Figure 4). The most common form of support provided by SRLs was provision of EQA panels, technical support, training and supervision visits.

### Barriers to implementation of a quality management system

**FIGURE 5:**
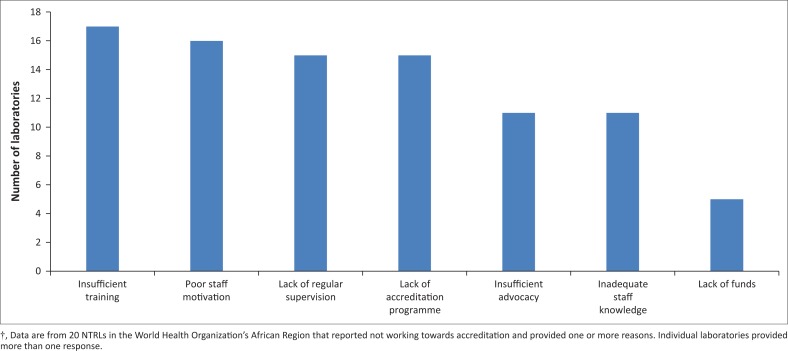
Reasons reported by National Tuberculosis Reference Laboratories in the World Health Organization African Region† for not currently working towards accreditation.

Of those laboratories not actively implementing QMS, the reasons for not having done so a QMS in their laboratory are shown in Figure 5. A lack of training, poor staff motivation, lack of an accreditation programme and insufficient on-site supervision were cited as the major reasons for non-implementation of a QMS. Interestingly, lack of funds was the lowest-rated response among the options provided in answer to this question. However, when laboratories were asked a separate question about the barriers to implementing QMS, lack of funds was cited as one of the main reasons. Furthermore, only 28.6% of respondees reported that they had included a budget and workplan for laboratory accreditation in their strategic plans or Global Fund concept notes.

## Discussion

### Recommendations

Although the majority of laboratories had knowledge of QMS tools, and almost half reported receiving SLMTA training or using the GLI tool, only a third of the laboratories reported having conducted an audit, an essential first step in establishing a baseline and developing an action plan to address recommendations and corrective actions resulting from the audit. Furthermore, responses from a number of countries suggested that there may be some confusion between the different tools mentioned in the survey. Since this survey did not provide a detailed measure of QMS implementation in individual laboratories and only a small proportion of laboratories were able to provide audit data, a full audit of laboratories using the SLIPTA checklist is an urgent priority in order to develop a detailed action plan and budget for achieving accreditation. It is also recommended that information on accreditation is disseminated to countries in the region explaining the process of accreditation, the tools available and an implementation roadmap is provided to assist countries with planning. Additionally, a regional database of suitably-qualified organisations and individuals who are able to provide technical assistance to countries for accreditation should be developed.

Participation in EQA for acid-fast bacilli smear microscopy was very high, with 95.8% of NTRLs participating in an EQA scheme. However, EQA for other diagnostic techniques was less frequently conducted, with 60.4% of NTRLs not participating in first-line DST EQA and very low participation in EQA for second-line DST and molecular methods. It is not known what proportion of the NTRLs conduct second-line DST, and therefore the low participation in EQA may, in some cases, reflect the fact the second-line DST is not currently conducted in the country. The same may apply to the low enrolment of NTRLs in molecular testing. Furthermore, the extent to which NTRLs perform EQA of laboratories within their network was not reported; it is these laboratories that conduct the majority of microscopy testing for patient care.

Participation in EQA for all NTRLs should be urgently expanded for all diagnostic technologies performed. This can be achieved by expanding access to EQA programmes through the SRL network or other recognised providers.

The majority of countries reported a formal agreement with an SRL. However, capturing of detailed information on the extent of support and to what extent the SRL is fulfilling the country’s need for technical support was outside the scope of the current survey. Establishment of formal relationships between the nine countries not currently supported by SRLs should be prioritised. Provision of EQA panels for all diagnostic technologies to all supported countries should be an essential component of the support provided by the SRL network or via other providers.

There was a disparity in QMS implementation and accreditation between Anglophone and non-Anglophone countries on the continent. This is anticipated to be due to the greater availability of tools and support in Anglophone countries in the region. Translation of any training materials or tools found to be lacking should be performed as an urgent priority, as well as building greater capacity for technical support in Francophone and Lusophone countries.

A lack of planning and budgeting were highlighted as major gaps, with almost three quarters of laboratories not having included plans for accreditation in their strategic planning processes. GLI Africa can play a role in providing guidance on planning and budgeting for accreditation, as well as advocating for the importance of quality improvement and accreditation as being key contributors toward countries meeting sustainable development goals for health. An assessment of country capacity and availability of partners and donors to support countries in planning, funding and implementing accreditation programmes should be conducted. Laboratories need to elicit senior management support within the Ministry of Health and include funding for quality improvement and accreditation of tuberculosis laboratories in their country strategic plans.^18^ Equally, donors need to allocate adequate funding for quality improvement and accreditation programmes. To ensure local political commitment, as well as continued and adequate donor funding, need more evidence on the benefit of accreditation. Such evidence should focus on the patient impact of improved quality and specifically the benefit of accreditation *per se* as opposed to quality improvement in the absence of accreditation, as well as including a costing and cost-benefit analysis.

### Limitations

The scope of this survey was to obtain a general overview of the knowledge and status of QMS among NTRLs on the continent, and not to provide detailed information about particular laboratories. Therefore, a clear indication as to the status of QMS implementation in individual laboratories was not possible as approximately two-thirds of laboratories had not been assessed using a recognised QMS checklist.

This survey focused on NTRLs and did not determine the status of QMS and progress toward accreditation for other laboratories in the network. In most countries, it would be anticipated that the NTRL is likely to be the most advanced in terms of QMS. It would be important to obtain information on the status of QMS in other laboratories, particularly those performing culture, DST and line-probe assay.

The survey was administered in three main *lingua franca* used in the African Region; namely, English, French and Portuguese. However, the WHO recognises other languages, including Spanish, as being the official language used in Equatorial Guinea. There were some conflicting responses given to several questions, implying that the questions were not clearly understood by all respondents. Finally, this survey was conducted among the 47 WHO African Region member states and not the 54 countries comprising the whole African region; therefore, generalisation of our conclusions to the entire African region should be made with caution.

### Conclusions

Progress has been made in implementing QMS in laboratories in the African Region. However, more than 90% of NTRLs in the region are not accredited. Nonetheless, a good foundation is in place on the continent from which to scale up laboratory accreditation efforts. The high level of participation in EQA for smear microscopy can be leveraged to expand the range of diagnostic tests covered by EQA. The majority of NTRLs reported having established a formal agreement with an SRL, although the scope and quality of support was not determined in this study. Building capacity for auditing is urgently needed, for both internal and external audit. Auditing of NTRLs is needed as a first step to inform individual country action plans and budgeting to achieve accreditation. Political commitment and strong leadership are needed to drive accreditation efforts; advocacy will require clear evidence of patient impact and cost-benefit to enable funding mobilisation from donors or governments among the many competing priorities for healthcare budgets. Dissemination of information on accreditation, coordination of technical support for planning, budgeting and implementing quality improvement and accreditation programmes is an urgent priority. Support for non-Anglophone countries should be strengthened, including translation of documents and tools, and provision of technical support in appropriate languages.
